# Effects of Virtually Led Value-Based Preoperative Assessment on Safety, Efficiency, and Patient and Professional Satisfaction

**DOI:** 10.3390/jcm14093093

**Published:** 2025-04-29

**Authors:** José Luis Gracia Martínez, Miguel Ángel Morales Coca, Marta del Olmo Rodríguez, Pablo Vigoa, Jorge Martínez Gómez, Jorge Short Apellaniz, Catalina Paredes Coronel, Marco Antonio Villegas García, Juan José Serrano, Javier Arcos, Cristina Caramés Sánchez, Bernadette Pfang, Juan Antonio Álvaro de la Parra

**Affiliations:** 1 Anesthesiology Department, General Villalba University Hospital, 28400 Madrid, Spain; 2 Information Technology and Systems Department, General Villalba University Hospital, 28040 Madrid, Spain; 3 Hospital Management, Quirónsalud 4-H Network, 28223 Madrid, Spain; 4 Clinical and Organizational Innovations Unit (UICO), Quirónsalud 4-H Network, 28223 Madrid, Spain; 5 Instituto de Investigación Sanitaria-Fundación Jiménez Díaz (IIS-FJD), 28015 Madrid, Spain; 6 Facultad de Ingenería Industrial, Universidad de Castilla La Mancha, 13071 Ciudad Real, Spain; 7 Hospital Management, Fundación Jiménez Díaz University Hospital, 28040 Madrid, Spain; 8 Coorporative Management, Quirónsalud Healthcare Network, 28040 Madrid, Spain

**Keywords:** anesthesiology, preoperative evaluation, preoperative assessment, preoperative care, quality improvement, value-based care

## Abstract

**Background:** The increasing demand for elective surgery makes optimizing preoperative assessment a priority. Value-based healthcare aims to provide the highest value for patients at the lowest possible cost through various mechanisms, including reorganizing care into integrated practice units (IPUs). However, few studies have analyzed the effectiveness of implementing virtually led IPUs for preoperative assessment. **Methods:** We performed a retrospective observational cohort study including patients undergoing elective surgery at a teaching hospital in Madrid, Spain from 1 January 2018 to 31 December 2023, analyzing changes in surgical complications, efficiency, and patient satisfaction between the pre-implementation (2018–2019) and post-implementation (2020–2023) periods. Anesthesiologists’ satisfaction with the virtual assessments was described. During the post-implementation period, preoperative assessment was reorganized as a virtually led IPU. At the IPU appointment, preoperative testing and physical (including airway) examinations were performed by a nurse anesthesiologist. The results were uploaded to the electronic health records, and asynchronous virtual anesthesiologist assessment using a store-and-forward approach was performed. Digital patient education was carried out over the Patient Portal mobile application. **Results:** A total of 40,233 surgical procedures were included, of which 31,259 were from the post-intervention period. During the post-intervention period, no increase in surgical complications was observed, while same-day cancellations decreased from 4.3% to 2.8% of the total procedures (*p* < 0.001). The overall process time did not increase, despite the rising number of surgical procedures per year. Patient satisfaction improved. The median time to complete anesthesiologist assessment was significantly lower for virtual assessment (4.5 versus 10 min (*p* < 0.001), signifying estimated time savings of 716 person-hours per year. Anesthesiologists agreed that virtual assessment was more efficient than in-person evaluation, and half of the participants agreed that virtual preoperative care improved their work–life balance and reduced burnout. **Conclusions:** A digitally enhanced value-based model of preoperative care can improve efficiency and satisfaction metrics, reducing unnecessary costs and potentially improving the quality of care.

## 1. Introduction

Preoperative assessment is a key component for ensuring safe, sustainable surgical care [1]. Pre-anesthesia evaluation clinics (PECs) for outpatient anesthetist-led preoperative assessment have demonstrated reductions in same-day surgical cancellations, surgical delays, and healthcare-related expenditure [2,3,4]. However, while PECs are increasingly common, the best model of PEC organization is unclear. The increasing demand for elective surgery in developed countries makes the optimization of preoperative assessment an international priority for anesthesiologists, surgeons, and healthcare managers [5,6].

Value-based healthcare emerged at the beginning of the century as a strategy to solve current problems in healthcare by focusing on delivering the highest value for patients at the lowest possible cost [7]. One of the main components of value-based care is the integration of care delivery by reorganizing care into integrated practice units (IPUs), in which different health professionals and services work together in the same physical space to provide patient-centered care and share the responsibility for costs and outcomes [8,9]. While IPUs have demonstrated improved patient satisfaction, reduced waiting times for patients, and better outcomes in other specialties [10,11,12,13], scarce literature exists on the implementation of integrated care delivery in preoperative care assessment.

On the other hand, the increasing importance of information and communications technology in medicine has led to a growing use of preoperative telemedicine [14]. This growth in the use of telemedicine was catalyzed by the COVID-19 pandemic and is present across all fields of medicine [15,16,17,18], with notable advantages, such as improved cost-efficacy measures, timely access to care, and availability to patients, albeit posing certain challenges inherent to each specialty. Digital interventions have proven to be a useful way of improving patient education prior to surgery and providing patient-centered care [19]. The available evidence indicates that virtual preoperative assessment is comparable to in-person evaluation regarding patient safety, patient experience, and efficiency [14,20,21]. However, the effectiveness of combining virtual care and IPUs in preoperative assessment has not been described.

This study evaluated the effects of redesigning an outpatient pre-anesthesia evaluation clinic in Madrid, Spain as a virtually led IPU. Using six years of data, we described changes in patient safety, patient satisfaction, and same-day surgical cancellations before and after implementation. We also performed a sub-analysis of efficiency metrics and healthcare professional experience, comparing virtual anesthesiology assessment with traditional, in-person evaluation.

## 2. Materials and Methods

### 2.1. Study Design

**Figure 1:**
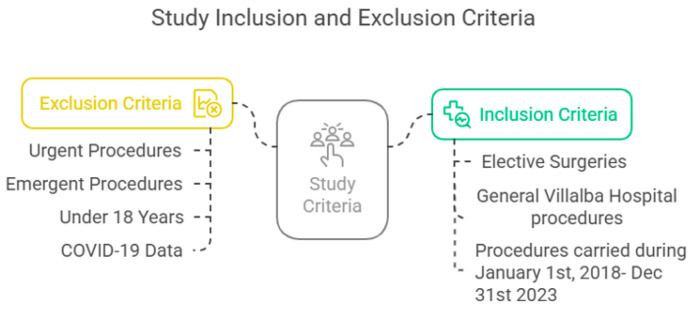
Study inclusion and exclusion criteria.

A retrospective before–after observational study was carried out, including pseudo-anonymized data from all elective surgical procedures performed at the General Villalba Hospital (Madrid, Spain) during a six-year period. The pre-implementation period was defined as 1 January 2018 to 31 December 2019. The post-implementation period was defined from 1 January 2020 to 31 December 2023. We excluded urgent and emergent procedures, as well as procedures taking place on patients younger than 18 years. Same-day cancellation data from 2020 were excluded to avoid bias related to the disruption caused by the COVID-19 pandemic. Figure 1 presents the inclusion and exclusion criteria for this study.

### 2.2. Intervention

**Figure 2:**
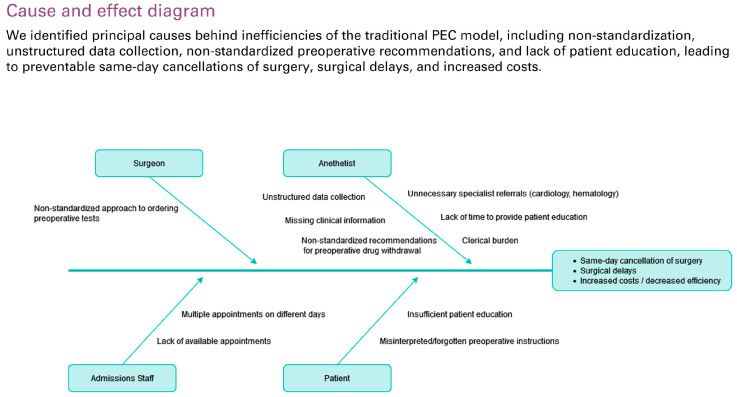
Cause-and-effect diagram analyzing principal inefficiencies of the traditional pre-anesthesia evaluation clinic (PEC) model.

**Figure 3:**
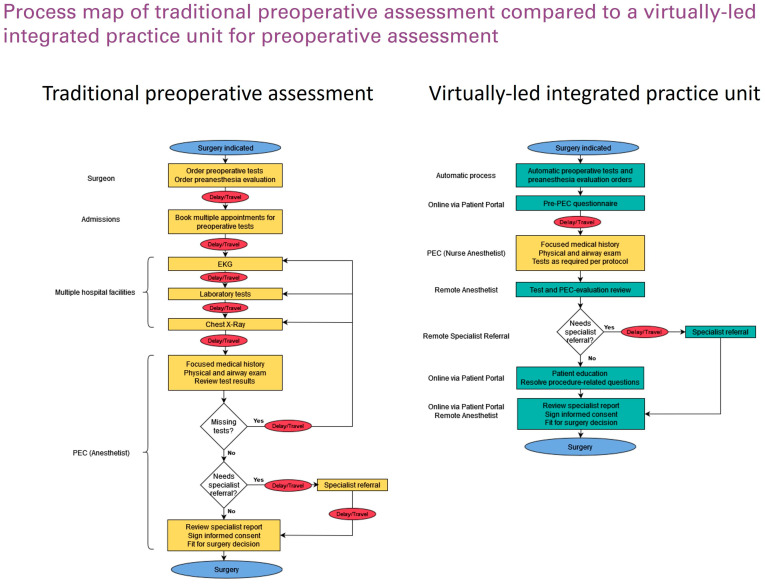
Process map comparing traditional preoperative assessment and a virtually led integrated practice unit for preoperative assessment.

The intervention was designed from September to December 2019 to address the study hospital’s increasing clinical burden of preoperative assessment. An anesthetist-led multidisciplinary team was formed to identify areas for improvement, including anesthesiologists, nurse anesthesiologists, members of the information technology and systems department, and hospital managers. Over a series of brainstorming sessions, “logistical pitfalls” along the preoperative patient journey were identified (Figure 2). To remediate these problems, a new patient workflow was designed, including a digital patient-administered questionnaire before the PEC appointment, an IPU with nurse-practitioner-led pre-anesthesia evaluation, and virtual anesthetist supervision for most patients (Figure 3). Before consultation, the patients fill out a pre-anesthesia questionnaire using a web app (the Quirónsalud Patient Portal), answering standardized questions about preexisting conditions and current medications.

A previously described algorithm was developed and integrated within the EHR to automatically place preoperative test orders based on surgical and patient characteristics [22]. The aim of the algorithm was to reduce unnecessary preoperative testing in line with patient safety initiatives such as “Choosing Wisely” [23,24]. We created an integrated practice unit (IPU) to unify testing and nurse-led pre-anesthesia evaluation on the same day, in the same space, reducing patient travel and wait times. Nurse anesthetists received specific training on focused clinical history and physical exams, using a checklist integrated with the EHR to ensure that all necessary items were covered. We developed a remote airway exam protocol (Supplementary S1), permitting virtual airway examination through a series of predetermined photographs uploaded to the EHR. At the end of the IPU encounter, all patients are discharged for virtual asynchronous (store-and-forward) anesthetist review. For patients discharged for virtual review, anesthetists review the nurse reports and test results over the EHR, and they either digitally approve patients for surgery or, exceptionally, order additional tests, specialist referrals, or an in-person anesthetist evaluation. The decision to refer patients for an in-person assessment is made based on anesthetists’ criteria, when one of more of the following motives are present: a suspected difficult airway requiring an in-person assessment, abnormal test results requiring further investigations, or in the case of the patient’s preference (the patient expressly requests an in-person evaluation with the anesthesiologist). Further patient education is provided through the Patient Portal, including educational videos on the benefits and potential risks of the type of anesthesia that will be used during their procedure. Patients are able to sign their informed consent electronically over the Patient Portal after completing the educational material.

Objectives and measurements
Primary objectives:

The primary objective of this study was to describe and analyze changes in surgical complications, efficiency metrics, and patient satisfaction after reorganizing the study hospital’s outpatient PEC as a virtually led IPU.

Safety metrics:

Surgical complications were used to estimate the effect of the initiative on patient safety. We analyzed the Agency for Healthcare Research and Quality Patient Safety Indicators (AHRQ-PSI) recorded as part of annual internal audit data (rates of postoperative bleeding, respiratory failure, pulmonary thromboembolism, deep vein thrombosis, sepsis, and wound dehiscence) for the pre- and post-implementation periods.

Efficiency measures:

Efficiency measures included in this study included rates of same-day cancellation and overall process times. To calculate the rates of same-day surgical cancellation, we excluded 2020 to avoid pandemic-related bias. Same-day cancellation data were taken from the hospital’s surgical database.

To calculate the process times, we randomly selected a sample of 381 patients from both groups, stratified by age and sex. The overall process time was defined as the time from the referral for preoperative evaluation to the date on which the patients underwent surgery. Sample size was calculated for a type I error of 0.05 and a type II error of 0.20, using MedCalc software (version 23.2.1).

Patient satisfaction:

To describe patient satisfaction, we analyzed differences in the NPS (Net Promoter Score) [25]. NPS surveys were randomly administered to patients attending the study hospital’s PEC during the pre- and post-intervention periods. Scores ranged from −100 to 100.

Secondary objectives:

The secondary objectives included describing demographic differences in the post-implementation period between patients undergoing virtually led assessment and those undergoing additional in-person anesthetist assessment. We also analyzed the differences in the time that the anesthesiologists took to complete virtually led and in-person assessments, as well as the differences in process time between the IPU encounter and anesthesiologist assessment when they were performed virtually and in-person, respectively.

To evaluate the anesthesiologists’ satisfaction with virtually led assessment, after performing a qualitative literature review to identify important aspects of clinician experience in virtual preoperative evaluation, we designed a 12-item survey, which was sent via email to anesthesiologists at the study hospital. The responses were recorded anonymously. The full survey is available in Supplementary S2. In this first assessment of professional satisfaction with the virtually led IPU, we focused on anesthetist experience. However, due to the crucial role of the nurse team, we expect to develop further analyses in which we include all professional staff involved in this project.

Statistical analysis:

Statistical analysis was performed using Python v 3.10. The Kolmogorov–Smirnov test was performed to determine the distribution of the different variables. Continuous variables were expressed as the mean (SD) or median (IQR), according to their distribution. Categorical variables were expressed as a number (percentage). The Mann–Whitney U test was used to compare differences in non-normal continuous variables between the study and control groups. The chi-squared test was used to compare differences in categorical variables. A *p*-value of <0.05 was deemed statistically significant.

Ethics and reporting standards:

This study was carried out according to the principles set forth in the Declaration of Helsinki. All data were pseudonymized. The study design was approved by the institutional ethics committee (Fundación Jiménez Díaz) on the 7 May 2024 (ethics approval code: EO084-24). We have included the document in the appendices (Supplementary S3)

Due to the retrospective nature of this study, the requirement for informed consent was waived. As a quality improvement initiative, the SQUIRE 2.0 guidelines [26] were followed when reporting this study (Supplementary S4).

## 3. Results

### 3.1. Comparison of Pre-Intervention and Post-Intervention Outcomes

During the pre-intervention period, a total of 8974 patients underwent preoperative assessment at the participating center, compared to 31,259 patients in the post-intervention period. Their median age was 58 years (27–84), and 46.5% of the patients were female. Differences in the demographic characteristics of patients between periods are summarized in Table 1.

Regarding patient safety, no differences in adverse perioperative events were observed between the two periods (Table 1). Patient satisfaction increased for the post-intervention period, from an NPS of 69.7 to 73.4. Same-day surgical cancelations decreased significantly from 4.3% to 2.8% of overall procedures in the post-intervention period (*p* < 0.001). No differences were observed in the overall process time, despite the increase in total surgical procedures per year.

### 3.2. Comparison of Virtual and In-Person Anesthesia Assessment

We performed a comparison of virtual and in-person anesthesia evaluation during the post-intervention period. The median age of patients in the virtual assessment group was almost 10 years younger than that of patients in the in-person group (53 years (21–81) versus 62 (20–84); *p* < 0.001). The time from the IPU visit to complete anesthesia evaluation was significantly shorter for patients in the virtual assessment group (4 (2–19) days versus 9 (2–25) days, *p* < 0.001).

Regarding anesthesiologists’ perspectives on the virtually led model of care, 8 (80%) out of 10 eligible consultant anesthesiologists answered the survey. The full results are presented in Table 2. Virtual evaluation was estimated to take a median of 4.5 min (range 2.5–10 min), while in-person assessment was estimated to take a median of 10 min (range 5–12 min), signifying estimated time savings of 716 person-hours per year. Discrepancies were observed regarding anesthesiologists’ perceptions of the quality of care provided by virtual care, while most participants agreed that virtual care improved efficacy. Half of the participants agreed or strongly agreed that virtual preoperative evaluation improved their work–life balance and reduced burnout. For example, some participants used their downtime during on-call hours to complete virtual assessments.

## 4. Discussion

This study analyzed six years of data from a teaching hospital in Madrid, Spain to describe changes in surgical safety metrics, efficiency, and patient satisfaction after implementing a virtually led value-based model of preoperative assessment. No increase in postoperative adverse events or overall process time was observed during the post-implementation period, despite increasing surgical volumes, while same-day cancellations decreased significantly and patient satisfaction scores for preoperative care improved. Upon analyzing post-implementation data, virtually led care was associated with lower process times and patient assessment times. The anesthesiologists generally agreed that virtual preoperative assessment increased efficiency and work–life balance, but opinions were divided as to whether virtual care influenced the quality of care and the clinician–patient relationship.

Many healthcare systems worldwide are investing in value-based care as a potential solution towards delivering high-quality, sustainable healthcare [27,28,29,30]. The principle behind value-based care is to provide the highest value to patients at the lowest possible cost [7]. In this regard, reorganizing preoperative care as a virtually led integrated practice unit has demonstrated several benefits for patients and healthcare professionals alike. On the one hand, this new model of care ensures that most patients scheduled for surgery only have to visit the hospital once in order to complete the required testing and physical examination, eliminating unnecessary travel and healthcare-related disruptions to their daily lives. Providing digital patient education over a secure mobile application prior to signing informed consent could theoretically improve patient engagement, which has demonstrated an association with improved clinical outcomes [31]. Likewise, asynchronous virtual anesthesiologist assessment following a “store-and-forward” model decreases the time that anesthesiologists have to dedicate to preoperative assessment by more than 50%, increasing the available operating room time and time for other professional activities, such as education and research. In this regard, half of the surveyed anesthesiologists agreed that virtual assessment improved their work–life balance and, interestingly, reduced professional burnout.

Since the COVID-19 pandemic, several initiatives featuring virtual preoperative assessment have been reported—for example, the latest European Guidelines of the European Society of Anesthesiology and Intensive Care Medicine (ESAIC)—for preoperative assessment in adults [32], demonstrating high overall patient acceptance and similar patient safety results compared to traditional, in-person care [14,33]. However, to the best of our knowledge, this is the first report of a store-and-forward approach to virtual anesthesiologist assessment. This report is in consonance with the aforementioned recommendations, which focus on telemedicine and standardized questionnaires as part of the preoperative anesthesia assessment to improve patient access to pre-anesthesia care and satisfaction [32]. At the same time, our approach gives a response to the need to expedite preoperative assessment, as explained throughout this article.

Patients responded favorably to the initiative, as demonstrated by increased Net Promoter Scores during the post-implementation period, while no increase in adverse postoperative events was observed. The importance of effective communication with patients is crucial to ensure high-quality care, and evidence-based strategies to ensure effective communication with patients and their carers should be implemented during preoperative assessment [34]. While patient satisfaction with clinician–patient communication was not directly evaluated in this study, it is probable that providing educational material over the Patient Portal application improved the patients’ knowledge about the different aspects of anesthesia. Most anesthesiologists agreed that the patients were satisfied with the virtual assessment. However, half of the respondents did not agree that the quality of virtual care was equal to that of in-person evaluation. The small sample size of respondents limits the validity of our findings, and future qualitative research is necessary to confirm and further explore these results.

It is necessary to recognize at this point that the implementation of the IPU has faced several challenges, including technical challenges with incorporating the virtual visit workflow in the electronic health record, as well as the need for professional training to implement the virtual assessment protocols. On the other hand, it was necessary to adapt the available physical space for the IPU. Finally, we initially faced some resistance to change from some staff members.

A notable challenge in virtual pre-anesthetic assessment is airway evaluation, which remains a critical limitation, particularly in accurately identifying potential difficult airways, as highlighted in recent studies [35,36]. Several factors contribute to this limitation, including the clinician’s experience and technical skills, the patient’s familiarity with online consultation platforms, and intrinsic patient characteristics that may predict a potentially difficult airway (PDA). However, the existing literature indicates a strong correlation between virtual and in-person assessments for non-PDA cases, suggesting that virtual consultations can be a reliable alternative in these scenarios [37].

Despite the volume of total surgical procedures per year almost doubling during the post-intervention period, no increase in overall process times was observed, which we hypothesize could be due in part to the increased efficiency of preoperative assessment after reorganization as an IPU. At the same time, a significant reduction in same-day cancellations was observed, indicating that the new model of care was successful in optimizing patient preparedness for surgery. Cancellation on the day of surgery is an important source of unnecessary healthcare expenditure and contributes to backlogs for elective procedures, with 10–20% of cancellations being due to inadequate preoperative workup [38,39,40,41]. Thus, we consider that implementing a virtually led IPU could increase value not only for patients but also for healthcare systems struggling to improve the efficiency of perioperative management and reduce surgical waiting times.

Our study has several limitations. Firstly, its retrospective nature and non-randomized design could potentially lead to biased results, specifically selection bias. However, data collection during the study period was performed homogeneously, and study variables such as patient safety data were calculated on a yearly basis, using official definitions by independent departments, reducing the risk of reporting bias. Also, the virtually led IPU was implemented for all patients during the second period of the study, so the changes in variables such as NPS can be attributed, at least in part, to the new model of care. At the same time, although we analyzed anesthesiologists’ experience, we did not include the perspectives of nurse practitioners operating the IPU, which represents a field for further research. Finally, the single-center nature of this study led to a relatively small sample size of participants answering the anesthetist satisfaction survey. While the answers to the survey are representative of the anesthetist team at the study center (80% response rate), further studies will be necessary in order to confirm these findings at a national or international level.

## 5. Conclusions

Reorganizing preoperative care as a virtually led IPU using a store-and-forward approach is associated with improved patient experience, reduced assessment times, and lower rates of same-day surgical cancellations, without negative effects on patient safety or overall process times, despite an increased volume of surgical procedures. A digitally enhanced value-based model of preoperative care can improve efficiency and satisfaction metrics, reducing unnecessary costs and potentially improving the quality of care. Further studies are necessary to evaluate anesthesiologists’ perceptions of the quality of virtual care compared to traditional, in-person preoperative assessment.

## Figures and Tables

**Table 1:** Differences in demographic characteristics, adverse preoperative events, patient satisfaction, and same-day cancellations between the pre-intervention and post-intervention periods.

	Pre-Intervention Period (2018–2019) N = 8974	Post-Intervention Period (2020–2021) N = 31,259	*p*-Value
Age, years (median (IQR)	57 (25–85)	59 (27–84)	0.003
Female sex (n, %)	4316 (48.1%)	14,382 (46.0%)	0.007
Postoperative bleeding *	18 (0.4%)	36 (0.3%)	0.83
Postoperative respiratory failure *	10 (0.3%)	19 (0.3%)	0.62
Pulmonary thromboembolism/deep vein thrombosis *	8 (0.2%)	15 (0.1%)	0.69
Postoperative sepsis *	1 (0.1%)	8 (0.1%)	0.56
Wound dehiscence *	4 (0.4%)	10 (0.4%)	0.92
Overall NPS	69.7	73.4	N/A
Same-day cancellations	384 (4.3%)	775 (2.8%) **	<0.001

* Agency for Healthcare Research and Quality Patient Safety Indicators (AHRQ-PSI), recorded as part of annual internal audit data (rates of postoperative bleeding, respiratory failure, pulmonary thromboembolism, deep vein thrombosis, sepsis, and wound dehiscence). For complete data, including sample size and inclusion criteria, see Supplement S2. ** Data from 2020 were excluded from analysis.

**Table 2:** Results of the survey on anesthesiologists’ perceptions of virtual preoperative assessment.

Question	Answer	N (%)
Sociodemographic data		
Sex	Female Male	3 (37.5%) 5 (62.5%)
Years working as a consultant in anesthesiology	0–2 3–5 5–9 >10	0 (0%) 0 (0%) 2 (25%) 6 (75%)
Years of experience in virtual preoperative assessment	1 2 3 >4	0 (0%) 0 (0%) 3 (37.5%) 5 (62.5%)
Quality, efficacy, and professional experience of virtual preoperative assessment		
Virtual assessment presents similar quality to in-person assessment	Completely disagree Somewhat disagree Neither agree nor disagree Somewhat agree Completely agree	3 (37.5%) 1 (12.5%) 0 (0%) 3 (37.5%) 1 (12.5%)
Virtual assessment is associated with high patient satisfaction	Completely disagree Somewhat disagree Neither agree nor disagree Somewhat agree Completely agree	0 (0%) 3 (37.5%) 0 (0%) 3 (37.5%) 2 (25%)
Virtual assessment reduces the quality of the doctor–patient relationship	Completely disagree Somewhat disagree Neither agree nor disagree Somewhat agree Completely agree	2 (25%) 1 (12.5%) 2 (25%) 1(12.5%) 2 (25%)
Virtual assessment improves process efficiency	Completely disagree Somewhat disagree Neither agree nor disagree Somewhat agree Completely agree	1(12.5%) 0 (0%) 1(12.5%) 2 (25%) 4 (50%)
Virtual assessment improves work–life balance	Completely disagree Somewhat disagree Neither agree nor disagree Somewhat agree Completely agree	1(12.5%) 1(12.5%) 2 (25%) 0 (0%) 4 (50%)
Virtual assessment reduces burnout	Completely disagree Somewhat disagree Neither agree nor disagree Somewhat agree Completely agree	2 (25%) 0 (0%) 1(12.5%) 2 (25%) 3 (37.5%)

## Data Availability

Underlying data not included in this article are available from the corresponding author upon reasonable request.

## Supplementary Materials

The following supporting information can be downloaded at https://www.mdpi.com/article/10.3390/jcm14093093/s1.
