# Supplementary material for: Effects of Virtually Led Value-Based Preoperative Assessment on Safety, Efficiency, and Patient and Professional Satisfaction
Source: J Clin Med. 2025 Apr 29;14(9):3093. doi: 10.3390/jcm14093093 (PMC12072373; doi:10.3390/jcm14093093)

Hospital General de Villalba

Virtual Airway Assessment

The objective of this document is to determine the characteristics of the photos that should be taken in the preoperative nursing consultation. The assessment/photographs that we will carry out are:

1. **Mallampati test**: we will ask the patient for a maximum opening of the oral cavity. We will stand at the same height as the patient to take the photograph. Hyperextension of the neck is not necessary to perform this test.

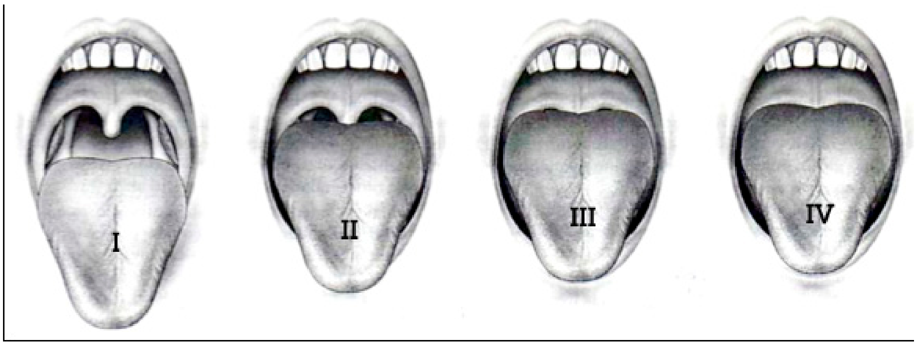

2. **Upper lip bite test**: we will ask the patient to bite their upper lip with their incisors and we will take the photograph.

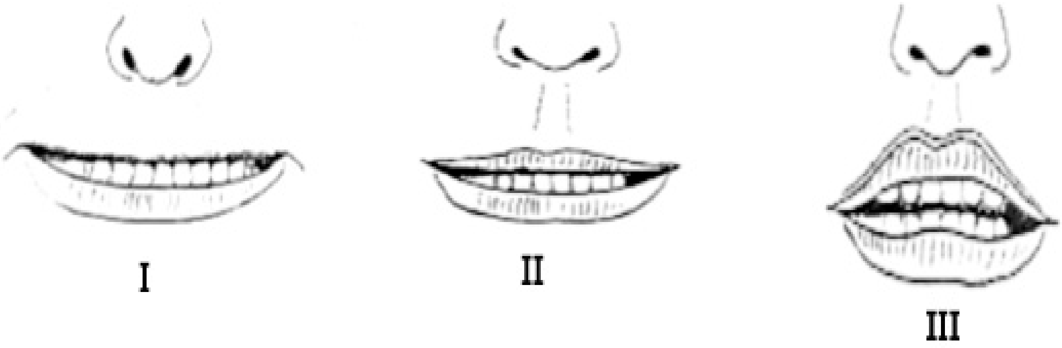

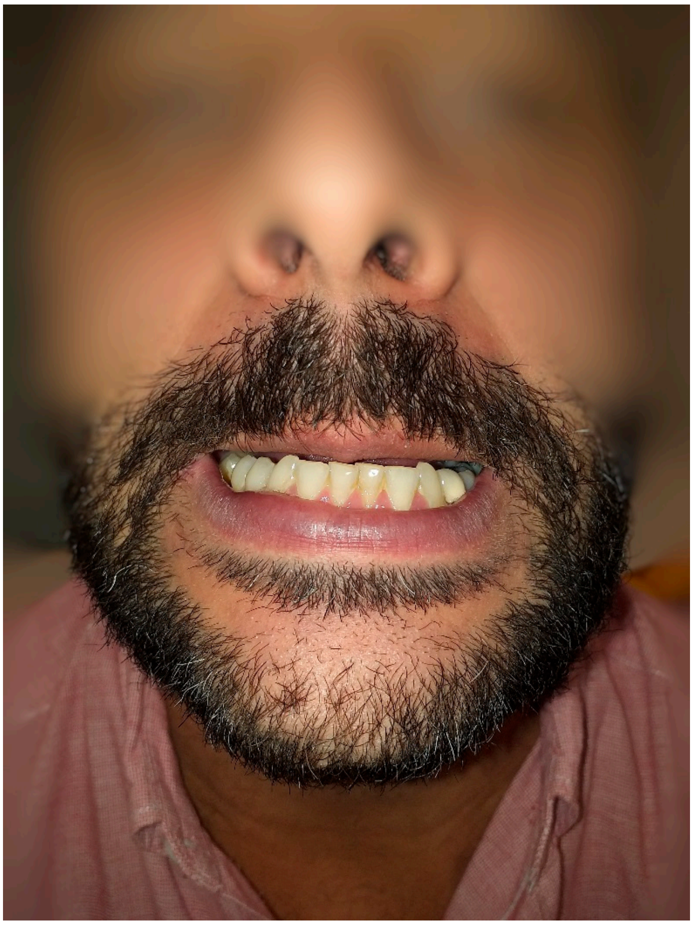

3. **Thyromental Distance (Patil Distance):** we will measure the distance between the mandibular symphysis and the thyroid protrusion, with the patient seated, the mouth closed and the neck in full extension. To take the photograph, we will ask the patient to hold a ruler from their chin to their thyroid cartilage, which they will point with the index finger of the other hand.

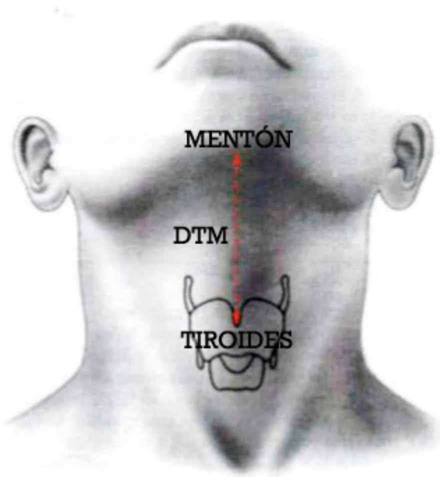
MENTÓN
DTM
TIROIDES

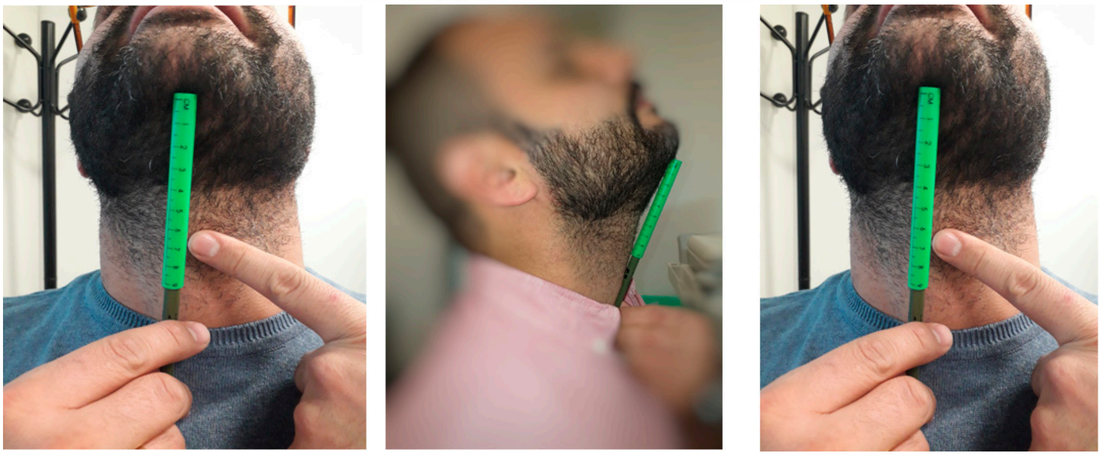

Supplement: Supplementary file 1 [file jcm-14-03093-s001.zip › Supplementary S1_English.pdf]
