# Supplementary material for: Effects of Virtually Led Value-Based Preoperative Assessment on Safety, Efficiency, and Patient and Professional Satisfaction
Source: J Clin Med. 2025 Apr 29;14(9):3093. doi: 10.3390/jcm14093093 (PMC12072373; doi:10.3390/jcm14093093)
Supplement: Supplementary file 1 [file jcm-14-03093-s001.zip › Supplementary S3.pdf]

INFORME DEL COMITE DE ÉTICA DE LA INVESTIGACION

Dra. Lucía Llanos Jiménez, Secretaria Técnica del COMITE DE ÉTICA DE LA INVESTIGACION DE LA FUNDACION JIMENEZ DIAZ

CERTIFICA:

Que en la reunión del CEIm-FJD que tuvo lugar el 26/03/2024 (acta nº 06/24) se evaluó el estudio referido y, ha decidido, una vez aceptadas las respuestas a las aclaraciones solicitadas:

A P R O B A R

La propuesta para que se realice el estudio:

Título: "Las Iniciativas de Salud Digital para Optimizar los Cuidados Preoperatorios: Estudio Observacional Retrospectivo Multicéntrico" .
Investigador Principal: JOSE LUIS GRACIA MARTINEZ. Hospital Universitario Fundación Jiménez Díaz
Servicio: Anestesiología
Promotor: UNIDAD DE INNOVACIÓN CLÍNICA Y ORGANIZATIVA RED 4-H
Código: DigitPC01
Documentos con Versiones:
PROTOCOLO Versión 2.0, del 22 de abril de 2024

Además, hace constar que:

1. En dicha reunión se cumplieron los requisitos establecidos en la legislación vigente –Decreto 39/94 de la CAM– para que la decisión del citado CEIm sea válida.

2. El Estudio reúne las normas éticas estándar de nuestra Institución para la realización de este tipo de estudios.

3. Se cumplen los preceptos éticos formulados en la Declaración de Helsinki de la Asociación Médica mundial sobre principios éticos para las investigaciones médicas en seres humanos y en sus posteriores revisiones, así como aquellos exigidos por la normativa aplicable en función de las características del estudio.

4. El CEImFJD, tanto en su composición como en sus procedimientos, cumple con las normas de BPC (CPMP/ICH/135/95) y con la legislación vigente que regula su funcionamiento, y que la composición del CEIm FJD es la indicada en el anexo I, teniendo en cuenta que en el caso de que algún miembro participe en el estudio o declare algún conflicto de interés no habrá participado en la evaluación ni en el dictamen.

5. Asimismo, hacemos constar que no existe contraprestación económica para el centro y los investigadores.

6. Además, este comité recuerda al Promotor (*) la obligación, en el caso de que se trate de un estudio prospectivo, de realizar el registro del estudio en una base de datos de acceso público antes de reclutar el primer paciente, así como el seguimiento del estudio de acuerdo con la legislación vigente.

(*) Para estudios promovidos por investigadores del IISFJD, se debe contactar con la Unidad de Investigación Clínica para información sobre cómo proceder para registrar el estudio (lucia.llanos@quironsalud.es)

Lo que firmo en Madrid a 07/05/2024

Dra. Lucía Llanos Jiménez
Secretaria Técnica CEImFJD

EO084-24_HRJC-HIE-HGV-FJD

Anexo I
COMPOSICIÓN DEL CEIm

Presidente:
JAVIER BECARES MARTÍNEZ (Farmacéutico - Hospital Universitario Fundación Jiménez Díaz)
Vicepresidente:
MACARENA BONILLA PORRAS (Farmacéutico - Hospital Universitario Fundación Jiménez Díaz)
Secretario:
LUCÍA LLANOS JIMÉNEZ (Farmacóloga - Miembro Comité de Investigación - Hospital Universitario Fundación Jiménez Díaz)
Vocales:
MIRIAM BLANCO RODRÍGUEZ (Médico Asistencial - Pediatría - Hospital Universitario Fundación Jiménez Díaz)
ALFONSO CABELLO UBEDA (Médico Asistencial - Medicina Interna - Hospital Universitario Fundación Jiménez Díaz)
RAÚL CÓRDOBA MASCUÑANO (Médico Asistencial - Hematología - Hospital Universitario Fundación Jiménez Díaz)
ANA GARCIA DIAZ (Abogado-a)
FRANCISCO JAVIER RUIZ HORNILLOS (Médico Asistencial. Miembro Comité de Investigación y Miembro del Comité de Ética Asistencial - Alergía - Hospital Universitario Infanta Elena)
OLGA SANCHEZ PERNAUTE (Médico Asistencial - Reumatología - Hospital Universitario Fundación Jiménez Díaz)
ANA DÍEZ ALCÁNTARA (Farmacéutico de Atención Primaria. Dirección Asistencial Noroeste)
SANDRA ZAZO HERNÁNDEZ (Bióloga Asistencial - Miembro Comité de Investigación - Anatomía Patológica - Hospital Universitario Fundación Jiménez Díaz)
ROSA SANCHEZ HERNANDEZ (Médico Asistencial - Nefrología - Hospital General de Villalba)
LORENA PINGARRON MARTÍN (Médico Asistencial - Cirugía Maxilofacial / Ortodoncia - Hospital Universitario Rey Juan Carlos)
MARTA MARIN CRESPO (Abogado-a)
FERNANDO ABELLAN-GARCIA SANCHEZ (Abogado-a)
MIGUEL MIR CORDERO (Lego no vinculado a la Institución)
CAROLINA GOTERA RIVERA (Médico Asistencial - Neumología - Hospital Universitario Fundación Jiménez Díaz)
MONTIEL JIMÉNEZ FUENTES (Médico Asistencial - Cirugía Digestivo / General - Hospital Universitario Fundación Jiménez Díaz)
ESTER CARREÑO SALAS (Médico Asistencial - Oftalmología - Hospital Universitario Fundación Jiménez Díaz)
ANGELA LAMARCA LETE (Médico Asistencial)
JOSE ANTONIO RUEDA CAMINO (Médico Asistencial - Miembro Comité de Investigación - Medicina Interna - Hospital Universitario Rey Juan Carlos)
CAROLINA MIRANDA CASTILLO (Médico Asistencial - Hematología - Hospital Universitario Rey Juan Carlos)
ROBERTO MARTIN REYES (Médico Asistencial - Cardiología - Hospital Universitario La Luz)
JOSE ANTONIO IGLESIAS BRAVO (DUE - Hospital Universitario Fundación Jiménez Díaz)

Anexo II

CENTROS E INVESTIGADORES PRINCIPALES Y COLABORADORES

Título: "Las Iniciativas de Salud Digital para Optimizar los Cuidados Preoperatorios: Estudio Observacional Retrospectivo Multicéntrico".
Investigador Principal: JOSE LUIS GRACIA MARTINEZ. Hospital Universitario Fundación Jiménez Díaz
Promotor: UNIDAD DE INNOVACIÓN CLÍNICA Y ORGANIZATIVA RED 4-H
Código: DigitPC01
Documentos con Versiones:
PROTOCOLO Versión 2.0, del 22 de abril de 2024

Fecha de actualización del anexo II: 07/05/2024

JOSE LUIS GARCIA MARTINEZ. Hospital General de Villalba.
LUIS ENRIQUE MUÑOZ ALAMEDA. Hospital Universitario Fundación Jiménez Díaz.
MARTA DEL OLMO RODRIGUEZ. RED 4-H.
JORGE SHORT APPELANIZ. RED 4-H.
BERNADETTE PFANG . RED 4-H.

C/ Isaac Peral, 42 Oficinas, . Oficina 1. 28015-Madrid
Teléfono: 91 5443720 – e-mail: ceic@fjd.es
